# Analysis for Reverse Temperature Dependence of Hydrogen Permeability through Pd-X (X = Y, Ho, Ni) Alloy Membranes Based on Hydrogen Chemical Potential

**DOI:** 10.3390/membranes10060123

**Published:** 2020-06-16

**Authors:** Asuka Suzuki, Hiroshi Yukawa

**Affiliations:** 1 Department of Materials Process Engineering, Graduate School of Engineering, Nagoya University, Furo-cho, Chikusa-ku, Nagoya 464-8603, Japan; 2 Department of Materials Design Innovation Engineering, Graduate School of Engineering, Nagoya University, Furo-cho, Chikusa-ku, Nagoya 464-8603, Japan; hiroshi@nagoya-u.jp

**Keywords:** hydrogen permeable membrane, palladium, phase transition, chemical potential, mobility

## Abstract

It is generally understood that the hydrogen permeability of Pd-Ag alloy membranes declines with decreasing temperature. However, recent studies have revealed that the hydrogen permeability of Pd-Ag alloy membranes inversely increases at a certain temperature range and reaches a peak. The peak behavior reflects the shape of pressure-composition isotherms (PCT curves). In order to elucidate the relationship between the reverse temperature dependence of hydrogen permeability and the PCT curves, the hydrogen permeability of pure Pd and Pd-X (X = Ho, Y, and Ni) alloy membranes were investigated. The pure Pd and Pd-5 mol%Ni alloy membranes, in which the α-α’ phase transition occurs, exhibits more significant peak behaviors than Pd-5 mol%Ho, Pd-5 mol%Y, and Pd-23 mol%Ag alloy membranes, in which the α-α’ phase transition is suppressed. Large differences in hydrogen solubility, at the hydrogen pressures above and below the plateau region or the inflection point, make the peak behaviors more significant. It is revealed that the peak temperature can be roughly predicted by the hydrogen pressure at the plateau regions or the inflection points in the PCT curves.

## 1. Introduction

Effective utilization of hydrogen energy is required to realize one of the sustainable development goals (SDGs) for 2030 adopted by the United Nations Summit, “SDG 7 affordable and clean energy” [1]. The hydrogen gas can be produced by various processes, including the steam reforming [2], dissolution reactions of biomass [3], and the electrolysis of water [4]. However, the produced gas always contains impurity gas other than hydrogen and needs to be purified before supplied to fuel cells [5]. Hydrogen permeable dense metallic membranes can separate and purify hydrogen gas with ultra-high purity [6]. Pd-based alloys such as Pd-Ag and Pd-Cu alloys are known as materials used for the hydrogen permeable dense metallic membranes [6,7]. Shirasaki et al. investigated the hydrogen permeability of various Pd-based alloy membranes at high temperatures [8]. The additions of Groups 3A, 1B, and 2B metals into Pd enhance the hydrogen permeability, whereas the additions of Groups 4A, 5A, 6A, 7A, and 8 metals decrease the hydrogen permeability [8]. Sakamoto et al. reported that Pd-Y and Pd-Ag-Y alloy membranes exhibit higher hydrogen permeability than pure Pd and Pd-Ag alloy membranes [9]. Piskin et al. investigated the hydrogen permeability of Pd-Ag-Ni alloy membranes by a combinatorial screening approach. The addition of Ni into Pd-Ag alloy membrane decreases the hydrogen permeability [10]. The Pd-Ag alloy membranes containing 20~25 mol% Ag are widely used practically for separating and purifying hydrogen gas from gas mixtures [6,7]. These Pd-Ag alloy membranes exhibit higher hydrogen permeability, compared with the pure Pd membrane [6]. The addition of Ag into Pd also improves the durability of the membrane by suppressing the α-α’ phase transition in the Pd-H binary system [11,12,13,14,15,16,17]. It has been considered that the Pd-Ag alloy membranes exhibit low hydrogen permeability at low temperatures [7] and cannot be applied below 300 °C. However, Suzuki et al. have recently reported that the Pd-Ag alloy membranes exhibit reverse temperature dependence of hydrogen permeability below 250 °C and achieve high hydrogen permeability comparable to 400 °C at 180 °C [16]. Also, it is revealed that the reverse temperature dependence is caused by the deviation of hydrogen solubility from the Sieverts’ law and can be understood by the consistent description of hydrogen permeation based on hydrogen chemical potential [18,19]. the Pd-based alloy membranes can be applied to the purification of hydrogen from gas mixture produced at low temperatures by utilizing this unique phenomenon by controlling the hydrogen solubility. 

In the present study, the reverse temperature dependence of hydrogen permeability for pure Pd and various Pd-based alloys, which contain elements to increase/decrease the hydrogen permeability of the Pd [8,9,10], were investigated. The hydrogen permeability was analyzed by the consistent description of hydrogen permeation based on hydrogen chemical potential [18,19], in order to evaluate hydrogen diffusivity and solubility separately. From these results, a concept is discussed to control the reverse temperature dependence of the hydrogen permeability.

## 2. Materials and Methods 

**Figure 1:**
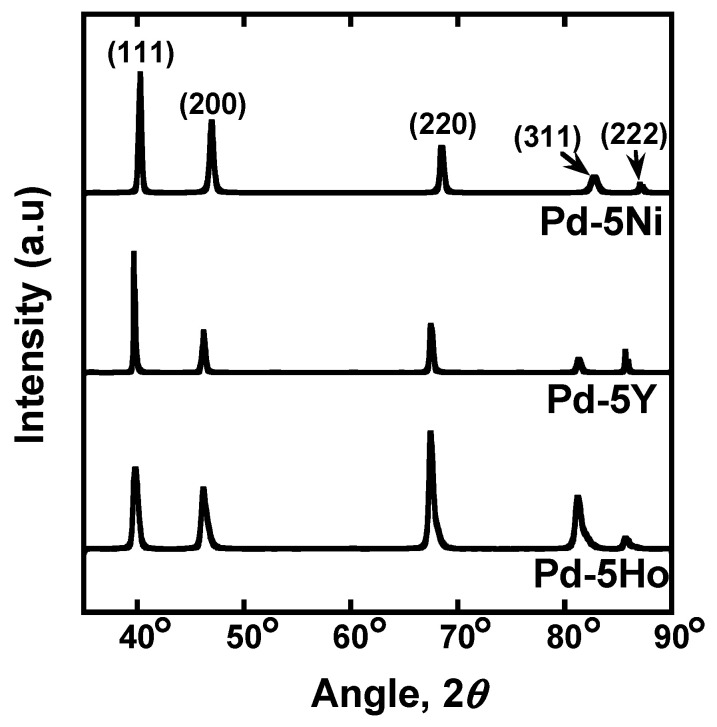
X-ray diffraction (XRD) profiles for Pd-5 mol%Ho, Pd-5 mol%Y, an Pd-5 mol%Ni alloy foils used in this study.

Pure Pd foil was prepared (Tanaka Kikinzoku Kogyo, Tokyo, Japan). Pd-5 mol% Ho, Pd-5 mol% Y, and Pd-5 mol% Ni alloy foils were fabricated by melting of raw materials and subsequent cold-rolling process. The thickness of each foil was 25 μm, 99 μm, 114 μm, 193 μm, respectively. In view of the equilibrium phase diagram for Pd-Ho, Pd-Y, and Pd-Ni binary systems [20,21,22], these foils are composed of a single phase with fcc crystal structure. Figure 1 shows the X-ray diffraction (XRD) profiles for Pd-5 mol% Ho, Pd-5 mol% Y, and Pd-5 mol% Ni alloy foils measured using Cu-Kα radiation operating at 40 kV and 40 mA (Rigaku Corporation, Tokyo, Japan). All the diffraction peaks are derived from the fcc crystal structure, in good agreement with phase diagrams. The foils were cut into disks with ϕ12 mm in diameter and annealed at 700 °C for 3600 s in a vacuum. The hydrogen permeation tests were carried out at 60°C~500°C by the gas permeation method applying pressure difference. The disk specimen was set into a cell for the hydrogen permeation test apparatus designed and constructed by ourselves and evacuated by a turbo molecular pump (TMP). Then, the sample cell was heated up to 500 °C while being evacuated. Subsequently, ambient pressure of air was introduced into the system and kept for 600 s. The sample cell was evacuated again, and then high purity (99.99999%) hydrogen gas was introduced into the system. A series of this oxidation and reduction process is known as “air-treatment” [23], which activates the sample surface for the hydrogen permeation. The hydrogen pressures at the feed and permeation sides of the membrane are controlled to be constant of 0.10 MPa, and 0.01 MPa, respectively. The hydrogen flux which permeates through the disk sample under the steady state condition was measured by monitoring the pressure change of a reserve tank with known volume. The temperature of the inner space of the reserve tank was monitored and confirmed to be almost constant during each hydrogen permeation test. Therefore, the pressure change can be converted into hydrogen flux by the following ideal gas law,

(1) $$J=\frac{\left(\Delta P_{3}/\Delta t\right)V}{RT}\cdot \frac{1}{S},$$

where (Δ*P*_3_/Δ*t*) is the pressure change of the reserve tank per unit time, *V* is the volume of the reserve tank, *R* is the gas constant, *T* is the absolute temperature of the reserved tank, *S* is the effective area of the membrane for hydrogen permeation. A detailed explanation of the hydrogen permeation test is given elsewhere [24]. Hydrogen flux was monitored firstly at 500 °C. After the steady-state hydrogen permeation reaction was confirmed at 500 °C, the hydrogen flux was evaluated. Subsequently, the temperature was lowered to the next measurement temperature, and the hydrogen flux was measured under the steady-state condition. These procedures were repeated to obtain the temperature dependence of hydrogen permeability. The hydrogen permeation coefficient was calculated by the following equation,

(2) $$\phi =\frac{J\cdot L}{P_{1}^{0.5}-P_{2}^{0.5}},$$

where *J* is the hydrogen flux through the sample foil, *L* is the thickness of the membrane, *P*_1_ and *P*_2_ are hydrogen pressures at the feed and permeation sides (i.e., 0.10 MPa, and 0.01 MPa, respectively). 

The pressure–composition isotherms (PCT curves) of pure Pd, Pd-5 mol% Ho, Pd-5 mol% Y, and Pd-5 mol% Ni were measured by a Sieverts–type apparatus (SUZUKI SHOKAN Co., Ltd., Tokyo, Japan) in order to investigate the hydrogen solubility. A small piece of the sample was put into a cell for the PCT apparatus and then evacuated. Subsequently, it was heated up to approximately 500 °C, followed by introducing high purity hydrogen gas (approximately 5 MPa). The sample cell was cooled down to room temperature under the hydrogen atmosphere, and evacuated again. This activation process was repeated 4 times prior to the PCT measurements. After the activation process, the PCT curves were measured at 400 °C and 250 °C up to about 2 MPa.

## 3. Results

**Figure 2:**
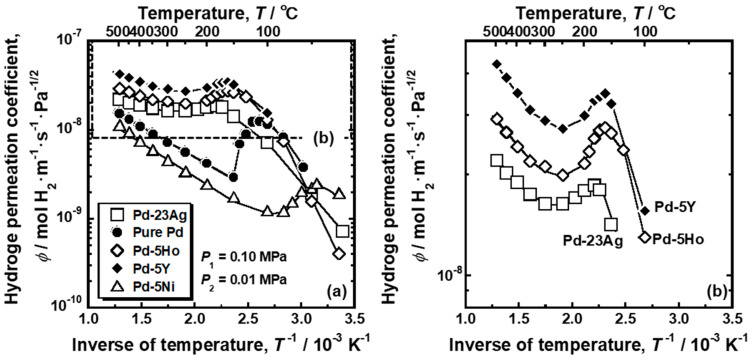
(**a**) Arrhenius plot of hydrogen permeation coefficient (*ϕ*) for pure Pd, Pd-5 mol% Ho alloy, Pd-5 mol% Y alloy, and Pd-5 mol% Ni alloy membranes. For comparison, hydrogen permeation coefficient for Pd-23 mol% Ag alloy membrane is also shown [18]. The results of Pd-5 mol% Ho, Pd-5 mol% Y, and Pd-23 mol% Ag alloys included in broken square are enlarged in (**b**).

Figure 2a shows Arrhenius plot of the hydrogen permeation coefficient (*ϕ*) of pure Pd, Pd-5 mol%Ho alloy, Pd-5 mol% Y alloy and Pd-5 mol% Ni alloy membranes. For comparison, the hydrogen permeation coefficient of Pd-23 mol%Ag alloy membrane is also shown in the figure [18]. The area enclosed by the broken line in Figure 2a for Pd-5 mol% Ho, Pd-5 mol% Y, and Pd-23 mol% Ag alloy membranes are enlarged and shown in Figure 2b. As shown in Figure 2b, the hydrogen permeation coefficient of Pd-5 mol% Ho, Pd-5 mol% Y, and Pd-23 mol% Ag alloy membranes decreased almost linearly with decreasing the temperature above around 300 °C, but it inversely increased below about 250 °C, and peaks were observed at around 160~180 °C. These alloy membranes exhibited almost the same peak behaviors, although the hydrogen permeability of Pd-5 mol% Y alloy membrane was the highest. As shown in Figure 2a, the hydrogen permeation coefficient of the pure Pd membrane decreased linearly with decreasing temperature above 150 °C but significantly increased below 150 °C. A peak was observed at approximately 110 °C. Note that the gradient of the temperature dependence of hydrogen permeability, through the pure Pd membrane, changed discontinuously at 150 °C, whereas the other alloy membranes exhibited continuous change from decrement to increment in hydrogen permeability with decreasing temperature. The Pd-5 mol% Ni alloy membrane also exhibited a large peak of the hydrogen permeation coefficient at approximately 45 °C, which was the lowest temperature among the alloys in this study. 

**Figure 3:**
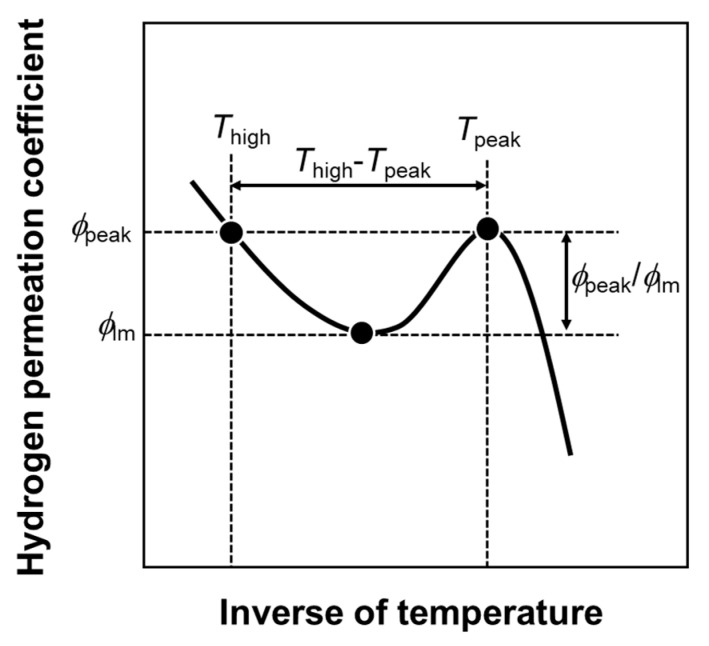
Schematic illustration of Arrhenius plot of the hydrogen permeation coefficient showing the definition of the values related to the peak behavior.

From Figure 2, values related to the peak behaviors were quantified. Figure 3 indicates the schematic illustration of Arrhenius plot of the hydrogen permeation coefficient showing the definition of the values related to the peak behaviors. The temperature at which the hydrogen permeation coefficient reaches its peak is denoted as the peak temperature (*T*_peak_). The hydrogen permeation coefficient at *T*_peak_ is defined as the peak hydrogen permeation coefficient (*ϕ*_peak_). In order to describe a height of the peak, the *ϕ*_peak_ is normalized by the local minimum of the hydrogen permeation coefficient (*ϕ*_lm_), which is denoted as the normalized peak hydrogen permeation coefficient (*ϕ*_peak_/*ϕ*_lm_). The higher temperature at which the hydrogen permeation coefficient reaches *ϕ*_peak_ (*T*_high_) is quantified to calculate the difference between *T*_high_ and *T*_peak_ (*T*_high_ − *T*_peak_) as an indicator of how much the operating temperature can be lowered while keeping the hydrogen permeability. 

The values related to the peak behaviors are summarized in Table 1. The peak temperatures (*T*_peak_) of the Pd-23 mol% Ag and Pd-5 mol% Ni alloy membranes were the highest (180 °C) and the lowest (45 °C) among the membranes investigated in the present study. The peak hydrogen permeation coefficient (*ϕ*_peak_) of the Pd-5 mol% Y alloy membrane was the highest (3.49 × 10^−8^ mol H_2_·m^−1^·s^−1^·Pa^−1/2^) and roughly 3 times higher than that of the pure Pd membrane. The normalized peak hydrogen permeation coefficient (*ϕ*_peak_/*ϕ*_lm_) of the pure Pd was approximately 4.3, indicating that the hydrogen permeation coefficient increased by roughly 4 times by the peak behavior. *ϕ*_peak_/*ϕ*_lm_ of the Pd-5 mol% Ni alloy membrane was the second highest. *ϕ*_peak_/*ϕ*_lm_ of the Pd-5 mol% Ho, Pd-5 mol% Y, and Pd-23 mol% Ag alloy membranes were almost the same values around 1.1~1.4. The pure Pd membrane exhibited the largest temperature difference between *T*_high_ and *T*_peak_ (*T*_high_ − *T*_peak_ = 330 °C), indicating that the operating temperature can be lowered the most while maintaining the hydrogen permeability. Also in the case of Pd-5 mol% Y, Pd-5 mol% Ho, and Pd-23 mol% Ag alloy membranes, *T*_high_ − *T*_peak_ was still large (220~290 °C), and the hydrogen permeability at 400~450 °C could be obtained even at 160~180 °C. Although the peak temperature (*T*_peak_) of Pd-5 mol% Ni alloy membrane was the lowest (approximately 45 °C), the hydrogen permeation coefficient at the peak temperature (*ϕ*_peak_) was the lowest and *T*_high_ − *T*_peak_ was the smallest. 

**Figure 4:**
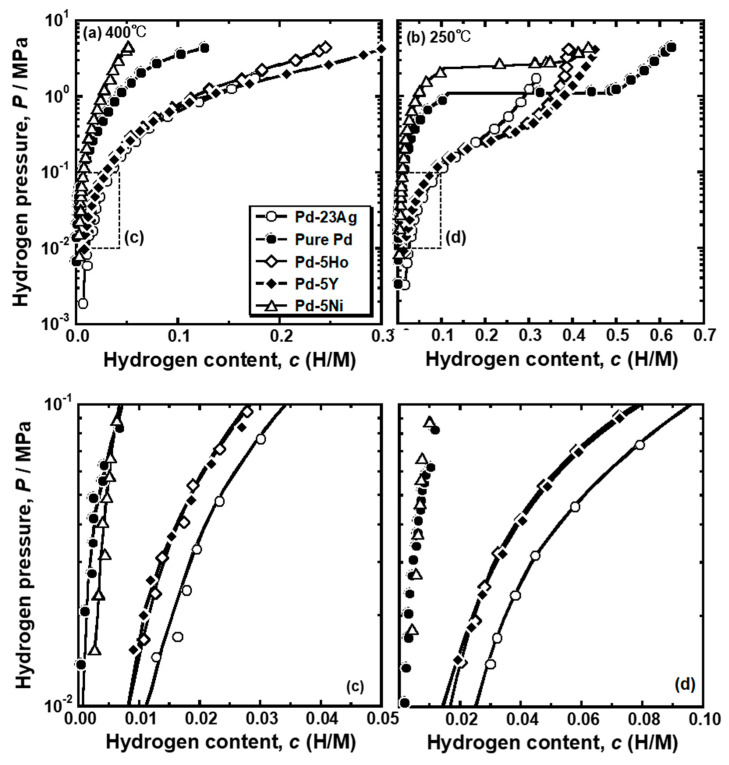
Pressure-composition-isotherms (PCT curves) for pure Pd, Pd-5 mol% Ho alloy, Pd-5 mol% Y alloy and Pd-5 mol% Ni alloy at (**a**) 400 °C and (**b**) 250 °C. For comparison, the PCT curves for Pd-23 mol% Ag alloy are also shown [18]. The PCT curves inside the broken lined squares in (**a**,**b**) are enlarged in (**c**,**d**).

Figure 4 presents the PCT curves of pure Pd, Pd-5 mol% Ho alloy, Pd-5 mol% Y alloy and Pd-5 mol% Ni alloys measured at (a) 400 °C and (b) 250 °C. The PCT curves for Pd-23 mol%Ag alloy are also drawn in the figure [16]. The hydrogen solubility was decreased by the addition of Ni into Pd, whereas the addition of Ho, Y, and Ag into Pd increased the hydrogen solubility. As shown in Figure 4b, the pure Pd and Pd-5 mol% Ni alloy showed clear plateau regions at 250 °C. The plateau region was caused by the α-α’ phase transition, which was predicted in the equilibrium phase diagram for Pd-H binary system. There were no clear plateau regions in the PCT curves of the Pd-5mol% Ho, Pd-5 mol% Y and Pd-23 mol% Ag alloys, suggesting that the α-α’ phase transition did not occur in these alloys. It is noted that the hydrogen concentrations in the pure Pd and Pd-5 mol% Ni alloy at higher pressure than the plateau pressure were higher than or almost equal to those in Pd-5mol% Ho, Pd-5 mol% Y and Pd-23 mol% Ag alloys. The PCT curves of the Pd-5 mol% Ho, Pd-5 mol% Y, and Pd-23 mol% Ag alloys were similar but slightly different. The PCT curves in the range of 0.01 MPa~0.10 MPa at each temperature were enlarged and shown in Figure 4c,d. The Pd-23 mol%Ag alloy exhibited slightly higher hydrogen solubility than the Pd-5 mol% Y and Pd-5 mol% Ho alloys in the range of 0.01 MPa~0.10 MPa. However, as shown in Figure 4b, the hydrogen solubility of Pd-23 mol% Ag alloy above 0.30 MPa at 250 °C was lower than the Pd-5 mol% Y and Pd-5 mol% Ho alloys. 

In order to evaluate the hydrogen diffusivity and solubility quantitatively, the following consistent description of hydrogen permeation based on hydrogen chemical potential was applied [19].

(3) $$J=\frac{RTB}{2L}\int_{c_{2}}^{c_{1}}c\frac{d\ln\left(P/P^{0}\right)}{dc}dc=\frac{RTB}{2L}f_{\mathrm{PCT}},$$

where *R* is the gas constant, *T* is absolute temperature, *B* is the mobility of hydrogen atoms, *c*_1_ and *c*_2_ are the hydrogen concentrations at the feed and permeation sides, and *P*^0^ is the standard hydrogen pressure (101325 Pa). The integral term in Equation (3) is defined as the PCT factor (*f*_PCT_) because it can be evaluated with the PCT curves [19]. 

**Figure 5:**
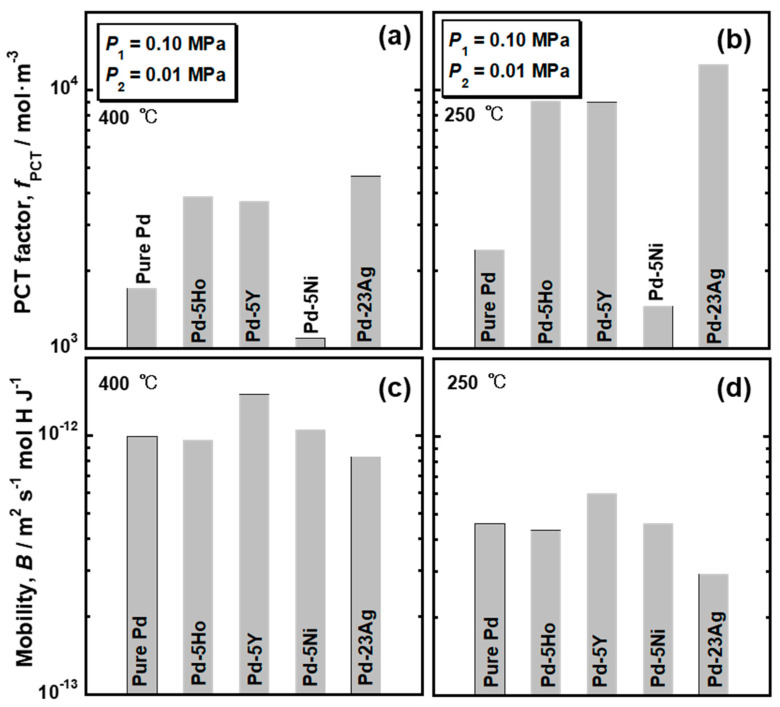
(**a**,**b**) PCT factors and (**c**,**d**) mobility of hydrogen atoms for pure Pd, Pd-5 mol% Ho alloy, Pd-5 mol% Y alloy, Pd-5 mol% Ni alloy, and Pd-23 mol% Ag alloy at (**a**,**c**) 400 °C and (**b**,**d**) 250 °C. The PCT factors are calculated under a pressure condition of 0.10 MPa at the feed side and 0.01 MPa at the permeation side.

The PCT factors of pure Pd and Pd alloys were quantified as the hydrogen solubility using the PCT curves in Figure 4 and the pressure condition (*P*_1_ = 0.10 MPa and *P*_2_ = 0.01 MPa). The mobility of hydrogen atoms (*B*) was also quantified by combining the results of the hydrogen permeation tests with the PCT factors. The PCT factors and mobility of hydrogen atoms at 400 °C and 250 °C are shown in Figure 5. Trends of the PCT factors and mobility of hydrogen atoms were almost similar at 400 °C and 250 °C although the absolute values were different depending on temperature. The PCT factors increased with decreasing temperature, while the mobility of hydrogen atoms decreased with decreasing temperature. The PCT factor of Pd-5 mol% Ni alloy was smaller than the pure Pd, whereas the addition of Ho, Y, and Ag into Pd improved the PCT factor. The addition of alloying elements exhibited slight effects on the mobility of hydrogen atoms at these temperatures. The hydrogen diffusivity in the Pd-5 mol% Ho and Pd-5 mol% Ni alloys was almost the same as the hydrogen diffusivity in the pure Pd. Pd-23 mol% Ag alloy exhibited lower hydrogen diffusivity than the pure Pd. The addition of Y into Pd enhanced the hydrogen diffusivity slightly. 

**Figure 6:**
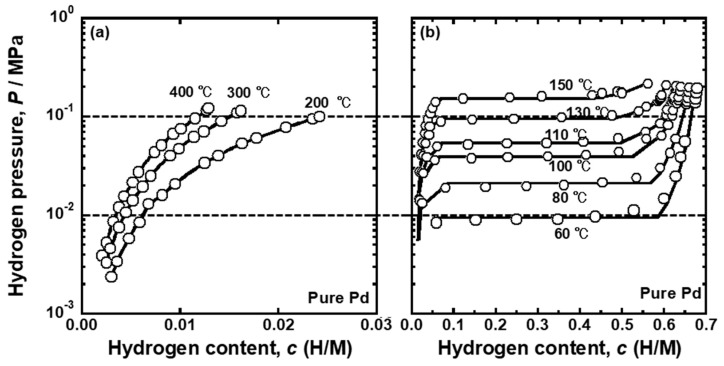
PCT curves for pure Pd at (**a**) 200~400 °C and (**b**)~150 °C reported in the literature [25].

**Figure 7:**
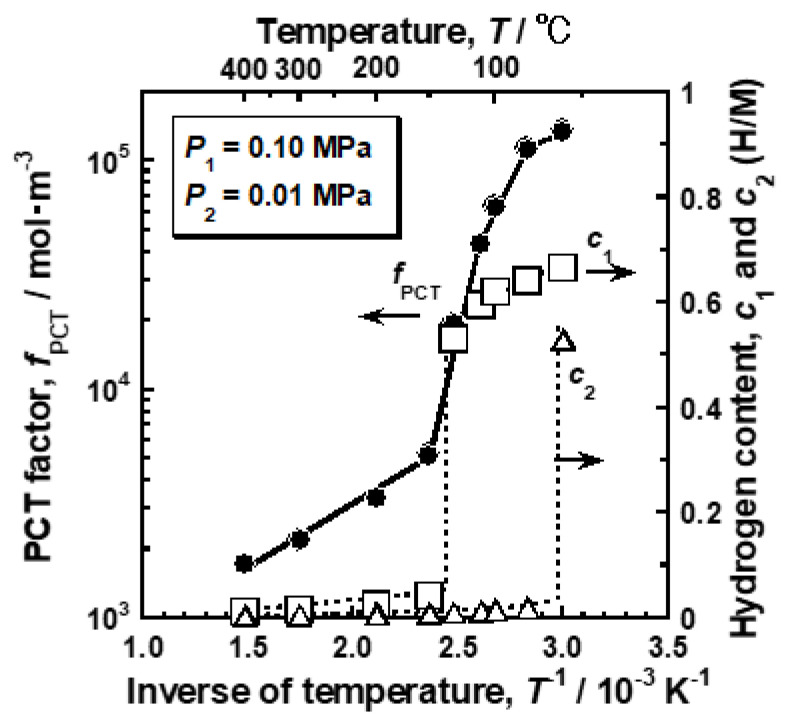
Changes in the PCT factor and hydrogen concentrations at feed and permeation sides of pure Pd as a function of the inverse of temperature. The PCT factor and hydrogen concentrations were quantified under a pressure condition of 0.10 MPa at the feed side and 0.01 MPa at the permeation side.

Figure 6 presents the PCT curves of the pure Pd reported in the literature [25]. The PCT curves were used for analyzing the temperature dependence of the PCT factor and mobility of hydrogen atoms for pure Pd. The horizontal broken lines in the figures indicate the pressure conditions applied for hydrogen permeation tests (*P*_1_ = 0.10 MPa and *P*_2_ = 0.01 MPa). As shown in Figure 6a,b, the plateau regions were not included in the pressure conditions above 150 °C. The pressure range of 0.01~0.10 MPa included the plateau region at 80~130 °C, indicating that the single α phase at high temperature becomes two phases of α/α’ as the temperature decreases during the hydrogen permeation tests. Below 60 °C, the specimen was composed of a α’ single phase under pressure condition of 0.01~0.10 MPa. In order to evaluate the PCT factor at the temperature where α and α’ phases co-existed, the following equation was used because the slope of the PCT curves (*d*ln(*P*/*P*^0^)/*dc*) is zero in the plateau region.

(4) $$f_{\mathrm{PCT}}=\int_{c_{2}}^{c_{1}}c\frac{d\ln\left(P/P^{0}\right)}{dc}dc=\int_{c_{2}}^{c_{L}}c\frac{d\ln\left(P/P^{0}\right)}{dc}dc+\int_{c_{H}}^{c_{1}}c\frac{d\ln\left(P/P^{0}\right)}{dc}dc,$$

where *c*_L_ and *c*_H_ are the equilibrium hydrogen concentration in α and α’ phase, respectively, under α/α’ two phase coexistence. Figure 7 shows the changes in the PCT factor (*f*_PCT_) and hydrogen concentrations at the feed and permeation sides (*c*_1_ and *c*_2_) as a function of the inverse of temperature. The PCT factor increased almost linearly with decreasing temperature above 150 °C. The PCT factor at 150 °C was roughly 3 times higher than that at 400 °C. When the temperature was below 150 °C, the PCT factor increased drastically. The decrease in temperature of only 20 °C from 150 °C provided approximately 4 times higher PCT factor. It is obvious that the significant increase in the PCT factor was caused by the α-α’ phase transition as the hydrogen concentration at the feed side increased drastically to approximately 0.5 (H/M) at 130 °C. When the α-α’ phase transition occurs, the plateau region does not contribute to the increase in the PCT factor because the slope of the PCT curves (*d*ln(*P*/*P*^0^)/*dc*) in the plateau region is zero. Therefore, the increase in the integral interval (*c*_2_~*c*_1_), due to the increase in *c*_1_, does not contribute much to the increase in the PCT factor. The significant increase in hydrogen concentration (*c*) included in the integral term rather caused the increase in the PCT factor. At 60 °C, the hydrogen concentration at the permeation side also increased drastically to approximately 0.5 (H/M), due to the α-α’ phase transition, resulting in the gradual increase in the PCT factor. 

**Figure 8:**
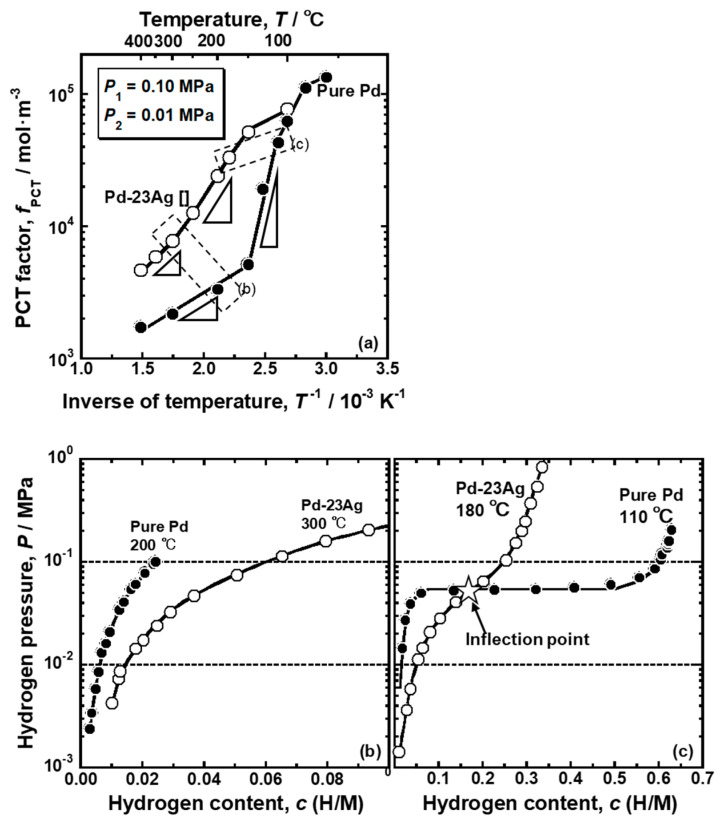
(**a**) Changes in the PCT factors of pure Pd and Pd-23 mol% Ag alloy as a function of the inverse of temperature. The PCT factors were quantified under a pressure condition of 0.10 MPa at the feed side and 0.01 MPa at the permeation side. (**b**,**c**) Corresponding PCT curves of the pure Pd and Pd-23 mol% Ag alloy at (**b**) 200 °C and 300 °C and (**c**) 110 °C and 180 °C (peak temperature).

The comparison of the PCT factor of the pure Pd and Pd-23 mol% Ag alloy is shown in Figure 8a. It is noticed here that the α-α’ phase transition does not occur in the Pd-23 mol% Ag alloy at around the peak temperature (180 °C) [18]. As shown in Figure 8a, both the pure Pd and Pd-23 mol% Ag alloy exhibited similar trend for the PCT factor to change along sigmoid curves. At higher temperature above 100 °C, the PCT factor of the Pd-23 mol% Ag alloy was higher than that of the pure Pd. The PCT factor of the pure Pd increased drastically around 150 °C, while the Pd-23 mol% Ag alloy increased largely around 300 °C. Also, the increment in the PCT factor of the pure Pd around 150 °C is much more significant than the increment in the PCT factor of the Pd-23 mol% Ag alloy around 300 °C. The difference of the changes in the PCT factors between the pure Pd and Pd-23 mol% Ag alloy could be easily understood by the PCT curves shown in Figure 8b,c. Since the Pd-23 mol% Ag alloy had a higher hydrogen solubility at high temperature than pure Pd (Figure 8b), it exhibited a higher PCT factor due to higher hydrogen concentration (*c*) and wider integral interval than pure Pd in Equation (3). The PCT curves of the Pd-23 mol% Ag did not have the plateau region even at low temperatures but instead had an inflection point (Figure 8c). The hydrogen pressure at the inflection point decreases with decreasing temperature. Here, the slope of the PCT curve is the smallest at the inflection point. Therefore, when the hydrogen pressure at the inflection point became lower than the hydrogen pressure at the feed side, the hydrogen concentration at the feed side increased drastically, resulting in a drastic increment of the PCT factor (Figure 8a). However, the increment was not as significant as the pure Pd with the plateau region because the hydrogen solubility above the inflection point in the PCT curve of the Pd-23 mol% Ag alloy was much lower than the hydrogen solubility in the α’ phase in the pure Pd (Figure 8c). In addition, due to higher hydrogen solubility in the Pd-23 mol% Ag alloy at high temperature (Figure 8b), the inflection point in the PCT curve of the Pd-23 mol% Ag alloy was included in the pressure condition at a higher temperature than the plateau region in the PCT curve of the pure Pd (Figure 8c). As a result, the PCT factor of the Pd-23 mol% Ag alloy increased drastically at higher temperature than pure Pd.

**Figure 9:**
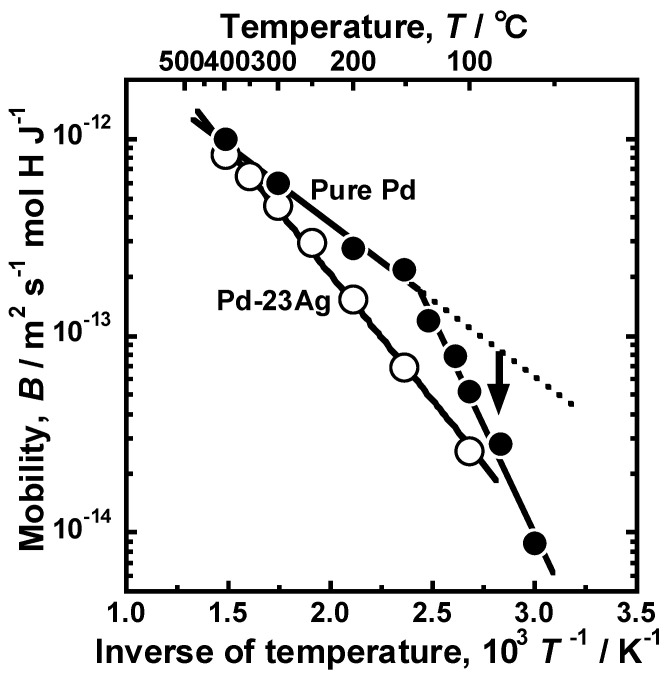
Arrhenius plot of the mobility of hydrogen atoms in pure Pd and Pd-23 mol% Ag alloy [18]. The broken line indicates extrapolation from the mobility at high temperature.

Figure 9 presents the Arrhenius plot of the mobility of hydrogen atoms in the pure Pd membrane. For comparison, the hydrogen mobility in the Pd-23 mol% Ag alloy membrane [18] is also shown in the figure. The temperature dependence of hydrogen mobility in the Pd-23 mol% Ag alloy membrane was described by the following Arrhenius equation as each plot almost aligns with a linear line in the figure.

(5) $$B=B_{0}\exp\left(-\frac{E}{RT}\right),$$

where *E* is the activation energy for hydrogen diffusion and *B*_0_ is the pre-exponential factor. In case of pure Pd membrane, although Arrhenius plot of the mobility of hydrogen was divided into two temperature regions at around 140 °C, the mobility of hydrogen atom also decreased almost linearly. Above 150 °C, the slope of the line of the pure Pd was smaller than that of the Pd-23 mol% Ag alloy, indicating that the addition of Ag into Pd increased the activation energy for hydrogen diffusion. However, the mobility of hydrogen atoms at lower temperatures below 130 °C deviated from the broken line extrapolated from the regression line at temperatures above 150 °C. The slope of the line, i.e., the activation energy for hydrogen diffusion increased below 130 °C, indicating that there are higher energy barriers for hydrogen diffusion in α’ phase. Also, the hydrogen mobility became almost half when the temperature decreases by only 20 °C from 150 °C. It is noted here that the mobility of hydrogen atoms decreased monotonically, even at the temperature range at which the hydrogen permeation coefficient increased drastically. 

## 4. Discussion 

In the present study, the effects of Ho, Y, and Ni additions into Pd on the reverse temperature dependence of the hydrogen permeability were investigated. The pure Pd membrane exhibited the most significant peak of the hydrogen permeability at approximately 110 °C (Figure 2). The gradient of the hydrogen permeation coefficient to the inverse of temperature at the local minimum point was discontinuous (Figure 2). The peak hydrogen permeation coefficient (*ϕ*_peak_) was approximately 4 times higher than the local minimum value of the hydrogen permeation coefficient (*ϕ*_lm_) (Table 1). The significant and discontinuous peak behaviors of the hydrogen permeability for pure Pd were caused by the α-α’ phase transition (Figure 5 and Figure 6). The hydrogen pressure at the feed side exceeds the plateau pressure, which decreases with decreasing temperature, provided the large hydrogen concentration at the feed side, resulting in much higher PCT factor (Figure 7). Although the mobility of hydrogen atoms decreased monotonically with decreasing temperature (Figure 9), the increment in the PCT factor (4 times from 150 °C to 130 °C) was far superior to the decrement in the mobility (half from 150 °C to 130 °C) (Figure 6 and Figure 8), leading to significant enhancement in the hydrogen permeability (Figure 2). 

The addition of Ag into Pd increased hydrogen solubility at high temperature and suppressed the α-α’ phase transition (Figure 8b,c), which provided moderate increase in the PCT factor at higher temperature, resulting in a moderate and continuous peak of hydrogen permeability (*ϕ*_peak_/*ϕ*_lm_ = 1.1) at higher peak temperature (*T*_peak_ = 180 °C) (Figure 2 and Table 1). The additions of Ho and Y into Pd have similar effects on hydrogen permeability to that of Ag into Pd (Figure 2 and Table 1): (1) Relatively moderate and continuous peak behaviors (*ϕ*_peak_/*ϕ*_lm_ = 1.1~1.4), and (2) higher peak temperature around 160~180 °C. 

On the other hand, the addition of Ni into Pd decreased the peak temperature to approximately 45 °C (Figure 2 and Table 1). Also, the Pd-5 mol%Ni alloy membrane exhibited a large peak (*ϕ*_peak_/*ϕ*_lm_ = 2.0) compared with the Pd-5 mol% Ho, Pd-5 mol% Y, and Pd-23 mol% Ag alloy membranes (Figure 2 and Table 1). These alloying effects on the hydrogen permeability will be discussed below. 

**Figure 10:**
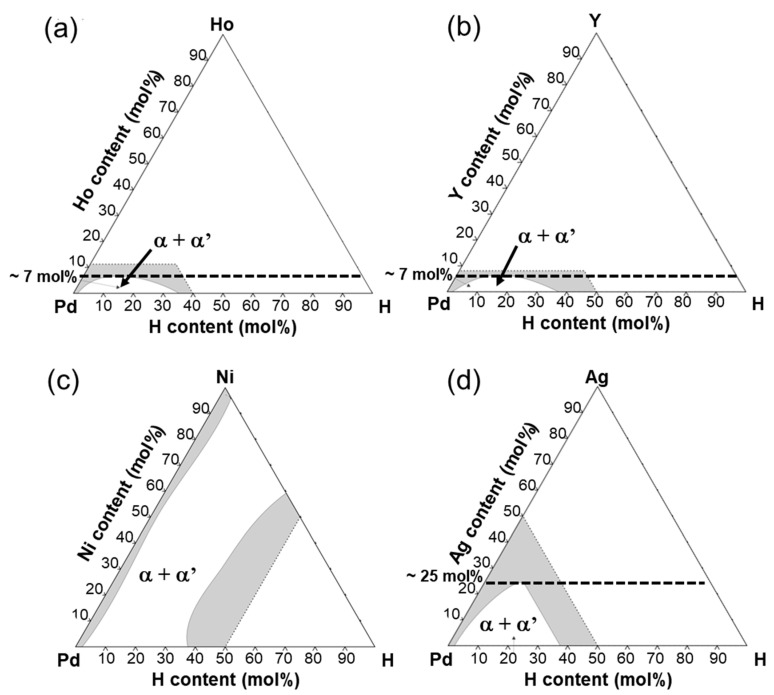
Isothermal sections at (**a**) 23 °C and (**b**–**d**) 25 °C in the equilibrium phase diagrams for Pd-H-X (X = (a) Ho, (b) Y, (c) Ni, (d) Ag) ternary systems [17,26].

Since the alloying elements in Pd slightly affected the mobility of hydrogen atoms (Figure 5c,d), the alloying effects on PCT factor dominantly contributed to the hydrogen permeability. It seems that the normalized hydrogen permeation coefficient (*ϕ*_peak_/*ϕ*_lm_) depends strongly on whether α-α’ phase transition occurs or not (Figure 2 and Figure 8a). Figure 10 shows the isothermal sections around room temperature ((a) 23 °C and (b-d) 25 °C) in the equilibrium phase diagrams for Pd-H-X (X = (a) Ho, (b) Y, (c) Ni, and (d) Ag) ternary systems [17,26]. The additions of Ho, Y, and Ag shrink the miscibility gap of α/α’ two phases (Figure 10a,b,d). The α-α’ phase transition is suppressed at room temperature by adding Ho and Y of approximately 7 mol% or Ag of approximately 25 mol%. In fact, no clear plateau region was observed in the PCT curves for Pd-23 mol% Ag and Pd-5 mol%X (X = Ho, Y) alloys even at 250 °C (Figure 4b), which is lower than the critical temperature of pure Pd (293 °C). It is also reported that the critical temperature (*T*_c_) of the α-α’ phase transition decreases almost linearly with increasing Ag concentration in Pd and was estimated to be approximately 20 °C in the case of Pd-23 mol%Ag alloy [18], which is lower than the peak temperature (180 °C). It is assumed here that the additions of Ho and Y into Pd also decreases the critical temperature of α-α’ phase transition linearly. Then, the critical temperature of the Pd-5 mol%Ho and Pd-5 mol%Y alloy is estimated to be approximately 100 °C, suggesting that the α-α’ phase transition did not occur at the peak temperature (160 °C). This suggestion is also supported by the continuous peak behavior similar to the Pd-23 mol% Ag alloy membranes as shown in Figure 2b. 

In contrast, as shown in Figure 10c, the addition of Ni into Pd hardly shrink the miscibility gap of α/α’ two phases, indicating that the α-α’ phase transition is not suppressed by the addition of Ni into Pd. In fact, Pd-5 mol% Ni alloy exhibited a clear plateau region in its PCT curve at 250 °C, resulting in hydrogen concentration higher than or almost equal to that of Pd-5 mol%Ho, Pd-5 mol% Y, and Pd-23 mol% Ag alloys. The larger peak of the hydrogen permeability of the Pd-5 mol%Ni alloy membrane compared with the Pd-5 mol% Ho, Pd-5 mol% Y, and Pd-23 mol% Ag alloy membranes (Table 1) is caused by the α-α’ phase transition. The hydrogen concentration in α’ phase in the Pd-5 mol%Ni alloy was reduced compared with the pure Pd (Figure 4), resulting in a lower normalized peak hydrogen permeation coefficient (*ϕ*_peak_/*ϕ*_lm_) (Table 1). Also, the increase in hydrogen solubility at low pressure range by the addition of Ho, Y, and Ag (Figure 3) decreases the normalized peak hydrogen permeation coefficient (*ϕ*_peak_/*ϕ*_lm_). The PCT factors of the Pd-5 mol%Ho, Pd-5 mol%Y, and Pd-23 mol%Ag alloys with high hydrogen solubility were higher than those of the pure Pd and Pd-5 mol%Ni alloy at higher temperatures than the peak temperature (*T*_peak_) (Figure 4), resulting in higher *ϕ*_lm_ and lower *ϕ*_peak_/*ϕ*_lm_. The peak temperature (*T*_peak_) is related to the plateau pressure or the hydrogen pressure at the inflection point of the PCT curve because the peak behaviors start from temperatures at which the plateau pressure or the inflection pressure is included in the pressure condition. Lower plateau pressure or hydrogen pressure at the inflection point causes the region or the point to be included in the pressure condition at higher temperatures (Figure 8c), resulting in a significant increase in the PCT factor at higher temperatures (Figure 8a). 

**Figure 11:**
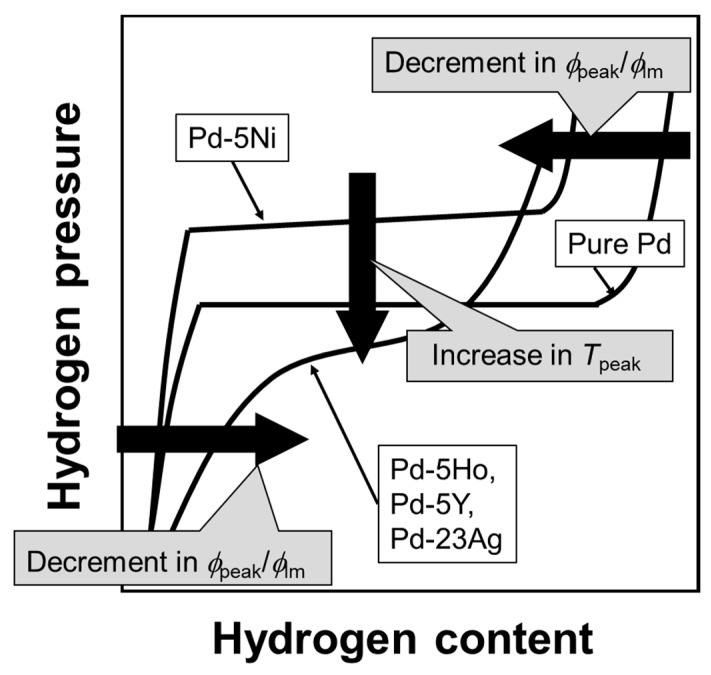
Schematic illustration of the PCT curves showing the relationship between the peak behaviors of the hydrogen permeability and the hydriding properties.

**Figure 12:**
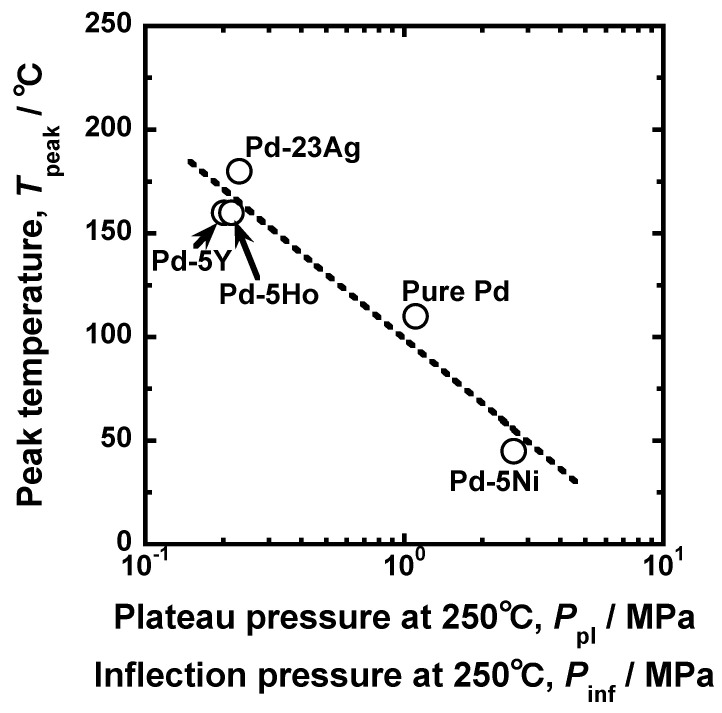
Relationship between the peak temperature and hydrogen pressure at plateau regions or inflection points in the PCT curves at 250 °C.

The relationship between the PCT curves and the peak behaviors of hydrogen permeability is schematically summarized in Figure 11. The higher/lower hydrogen solubility above/below the plateau region or the inflection point enhances the normalized hydrogen permeation coefficient (*ϕ*_peak_/*ϕ*_lm_). Lower plateau pressure or hydrogen pressure at the inflection point increases the peak temperature (*T*_peak_). Figure 11 suggests that the peak behaviors can be predicted from the PCT curves at higher temperatures than the peak temperature. Figure 12 presents the relationship between the peak temperature and the hydrogen pressure at the plateau regions or the inflection points in the PCT curves at 250 °C. There is a linear correlation between the peak temperature and the hydrogen pressure at the plateau region or the inflection points in the PCT curves, indicating that the peak temperature can be roughly predicted by the PCT curves at 250 °C, i.e., a temperature higher than the peak temperature. 

In order to make the peak behaviors more significant at lower temperatures, it is effective to increase both the plateau pressure and hydrogen solubility at α’ phase. On the other hand, suppression of the α-α’ phase transition is practically important because the α-α’ phase transition declines the durability of the membrane by crystal lattice expansion [27]. Therefore, the addition of elements like Ho, Y, and Ag into Pd is effective for the practical utilization of the peak behaviors. In particular, Pd-5 mol% Y alloy membranes exhibited approximately 2 times and 1.5 times higher peak hydrogen permeation coefficient than the Pd-23 mol% Ag and Pd-5 mol% Ho alloy membranes (Table 1). The hydrogen concentration above the infection point in the PCT curve of the Pd-5 mol% Y alloy was higher than those of the Pd-5 mol% Ho and Pd-23 mol% Ag alloys (Figure 4b), leading to higher PCT factor at the peak temperature. Also, the hydrogen mobility in the Pd-5 mol% Y alloy was a little higher than those in the Pd-5 mol% Ho and Pd-23 mol% Ag alloys (Figure 5c,d). Both higher PCT factor and mobility of hydrogen atoms contribute to the higher peak hydrogen permeation coefficient. One of the possible reasons for higher hydrogen solubility above the inflection point and hydrogen mobility maybe that Y is an element having high affinity for hydrogen (Enthalpy for hydrogen dissolution: −82 J·mol^−1^ [28]) d large atomic size (1.773 Å [29]). It is considered that the strong affinity for hydrogen of Y increases the hydrogen solubility at high hydrogen concentration by the preferential occupation of hydrogen near Y atoms. The large atomic size of Y in Pd expands the face-centered cubic (fcc) crystal lattice. Recently, Kimizuka et al. reported that the expansion of Pd lattice stabilizes the tetrahedral sites and enhance the hydrogen diffusivity [30]. The lattice expansion by the addition of Y might provide such an effect of the hydrogen diffusivity. On the other hand, Ho also have a large atomic size (1.767 Å [29]) although the addition of Ho hardly changed the mobility of hydrogen atoms (Figure 5c,d). The interactions between Y and hydrogen atoms and between Ho and hydrogen atoms need to be investigated in future studies. 

Even when the α-α’ phase transition is suppressed, the peak behaviors are accompanied by a significant increase in hydrogen concentration at the feed side, which will lead to crystal lattice expansion. When the operation of the membranes is thermally cycled, there is a possibility to decline the durabilities due to the repeated lattice expansion and shrinkage. Also, in the present study, the peak behaviors under pure hydrogen gas atmosphere were investigated. However, the presence of impurity gases such as H_2_S, CO, CO_2_, and H_2_O may affect the peak behaviors. Therefore, the stability of the membrane under thermal cycling and the presence of impurity gases need to be investigated in the future. 

Another important finding is that the activation energy for hydrogen diffusion increases due to the α-α’ phase transition (Figure 9). There are three possible reasons for inhibiting hydrogen diffusion: (1) Occupation of neighboring interstitial sites, (2) ordering of hydrogen configuration, and (3) formation of the superabundant vacancies. The α-α’ phase transition provides higher hydrogen concentration above 0.5 (H/M). In fcc crystal structure, hydrogen atoms occupy octahedral sites [28]. There is one octahedral site per one metal atom, indicating that the maximum hydrogen concentration is structurally 1.0 (H/M). Hydrogen concentration above 0.5 (H/M) indicates that half of the octahedral sites toward which a hydrogen atom jumps are already occupied by other hydrogen atoms. However, by occupations of neighboring interstitial sites, the pre-exponential factor for hydrogen diffusion is likely to decrease rather than the activation energy increases. The increase in the activation energy for hydrogen diffusion is caused by the stabilization of hydrogen atoms in interstitial sites. It is known that hydrogen atoms repulsively interact with each other in metals at high hydrogen concentration state [28], leading to not occupying interstitial sites at the first nearest neighbor of the occupied sites. It seems that hydrogen atoms are ordered to keep the distance from each other [28]. Extra energy is required for hydrogen atoms to diffuse, while disturbing the ordered configuration, and the activation energy might increase. In addition, it is known that the α-α’ phase transition (hydride formation) generates superabundant vacancies [31]. Hydrogen atoms are trapped in the vacancies with potential valleys deeper than interstitial sites. The activation energy increases because higher thermal energy is required to escape from such vacancies. Further investigations will be needed to elucidate a dominant contributor for increasing the activation energy, due to the α-α’ phase transition by systematical permeation tests, first principles calculations, positron annihilation, neutron scattering, and so on. 

## 5. Conclusions 

In the present study, the reverse temperature dependence of hydrogen permeability through pure Pd and various Pd-based alloy membranes is investigated. The pure Pd membranes exhibites a significant and discontinuous peak behavior, which is caused by the α-α’ phase transition. Although the activation energy for hydrogen diffusion increases by the α-α’ phase transition, significant increment in the PCT factor is far superior to the decrement in the mobility of hydrogen atoms. The addition of Ho, Y, and Ag into Pd suppress the α-α’ phase transition, resulting in moderate and continuous peak behaviors. The addition of Ni into Pd does not contribute to the suppression of the α-α’ phase transition and generates a large peak at the lowest temperature. It is revealed that the peak behaviors can be roughly predicted by the PCT curves at a temperature higher than the peak temperature. Among the alloys investigated in the present study, the Pd-5 mol%Y exhibits the most preferred peak behaviors. The peak is not due to the α-α’ phase transition and shows the highest peak hydrogen permeation coefficient.

## Figures and Tables

**Table 1:** Peak temperature (*T*_peak_), hydrogen permeation coefficient at *T*_peak_ (*ϕ*_peak_), normalized hydrogen permeation coefficient at *T*_peak_ (*ϕ*_peak_/*ϕ*_lm_), higher temperature to obtain the same hydrogen permeation coefficient as *ϕ*_peak_, (*T*_high_), and the difference between *T*_peak_ and *T*_high_ (*T*_high_ − *T*_peak_) for pure Pd, Pd-5 mol%Ho alloy, Pd-5 mol%Y alloy, Pd-5 mol%Ni alloy and Pd-23 mol%Ag alloy.

Material	*T*_peak_/°C	*ϕ*_peak_/mol H_2_·m^−1^·s^−1^·Pa^−1/2^	*ϕ*_peak_/*ϕ*_lm_	*T*_high_ − *T*_peak_/°C
Pure Pd	110	1.25 × 10^−8^	4.3	330
Pd-5Ho	160	2.76 × 10^−8^	1.4	290
Pd-5Y	160	3.49 × 10^−8^	1.3	240
Pd-5Ni	45	0.25 × 10^−8^	2.0	155
Pd-23Ag	180	1.85 × 10^−8^	1.1	220
